# Aberrant DNA methylation of synaptophysin is involved in adrenal cortisol-producing adenoma

**DOI:** 10.18632/aging.102119

**Published:** 2019-07-28

**Authors:** Jia-Yu Zhong, Rong-Rong Cui, Xiao Lin, Feng Xu, Ting Zhu, Fuxingzi Li, Feng Wu, En Zhou, Lu Yi, Ling-Qing Yuan

**Affiliations:** 1 Department of Metabolism and Endocrinology, National Clinical Research Center for Metabolic Diseases, The Second Xiang-Ya Hospital, Central South University, Changsha, Hunan, People’s Republic of China; 2 Department of Geriatrics, Institute of Aging and Age-related Disease Research, The Second Xiang-Ya Hospital, Central South University, Changsha, Hunan, People’s Republic of China; 3 Department of Pathology, The Second Xiang-Ya Hospital, Central South University, Changsha, Hunan, People’s Republic of China; 4 Department of Otorhinolaryngology Head and Neck Surgery, Hunan Provincial People’s Hospital, Changsha, Hunan, People’s Republic of China; 5 Department of Urology, The Second Xiang-Ya Hospital, Central South University, Changsha, Hunan, People’s Republic of China

**Keywords:** synaptophysin, DNA methylation, adrenal cortisol-producing adenoma, microRNA

## Abstract

Cortisol-producing adenoma (CPA) is the main cause of Adrenal Cushing syndrome. However, its molecular mechanism is not fully understood. Previous study revealed Synaptophysin (*SYP*) is ubiquitously expressed in adrenocortical tumors, but its function in CPA still need to be discovered. In the present study we determine the molecular mechanism involved in *SYP* dysregulation in CPA and how *SYP* affects the secretion of cortisol in CPA. Our results showed that aberrant DNA methylation of *SYP* is involved in CPA progress. Using a miRNA microarray and qRT-PCR, we found decreased expression of miR-27a-5p in CPA compared with normal adrenal tissue. Moreover, the expression of TET3, the target gene of miR-27a-5p, increased in CPA compared with normal adrenal tissue. Knock-down of TET3 resulted in hypermethylation of SYP which reducing the expression level of SYP in H295R cells. The miR-27a-5p-TET3-SYP signalling pathway may regulate proliferation and cortisol secretion in H295R cells and, thus, play a key role in CPA development.

## Introduction

Adrenal Cushing syndrome (CS), consists of a set of systemic manifestations similar to those found in aging, is caused by glucocorticoid excess due to a cortisol-producing adenoma (CPA) or adrenocortical carcinoma [1]. It is associated with hypertension, physical and cognitive degeneration in aging and accelerated atherogenesis, obesity, and osteoporosis [2–4]. Hypersecretion of cortisol from the adrenal cortex is a character of CPA. Moreover, hypercortisolism might  negatively impact telomere maintenance and consequently to premature aging [5,6]. However, the mechanism involved in this hyperfunction of the adrenal cortex in CPA remains unknown**.**

Synaptophysin (*SYP*; molecular weight, 38-kDa) is an integral membrane protein and a neuroendocrine marker [7] involved in synaptic vesicle formation [8], and the *SYP* gene family is involved in neuronal and neuroendocrine differentiation in rats and humans [9,10]. The adrenal cortex is not an intrinsic part of the diffuse neuroendocrine system, but neuroendocrine differentiation appears in some adrenocortical tumours [11]. Previous studies reported that *SYP* is ubiquitously expressed in adrenocortical tumours and its expression in adrenocortical adenomas may be associated with functions such as transport or secretion of glucocorticoids [12]. Hence, it is possible that *SYP* may play an important role in adrenocortical adenoma tissues. However, the effect of *SYP* and the mechanisms of the *SYP* genomic or genetic alterations in CPA still need to be validated.

One of the most important epigenetic modifications of the genome is DNA methylation, which occurs on cytosine residues at carbon 5 of the pyrimidine ring of simple sequences termed CpG dinucleotides, and subsequently controls gene expression. DNA methylation at CpG islands is strongly related to stable transcriptional repression [13,14]. Thus, DNA methylation regulates expression of many genes and is involved in many human diseases. Recently, aberrant global and gene-specific DNA promoter methylation has been observed in human adrenocortical tumours [15], implicating dysregulation of steroid biosynthesis. Thus, we hypothesized that methylation of cytosine nucleotides in CpG islands of the *SYP* promoter may regulate *SYP* gene expression and contribute to the hyperfunction of the adrenal cortex in CPA.

microRNAs are small RNA molecules that regulate gene expression by a posttranslational repression mechanism and are predicted to target up to 30% of the mammalian genome [16,17]. These molecules play important roles in various biological processes, including cell proliferation, differentiation, apoptosis and migration [18,19]. Accordingly, numerous studies have suggested that aberrant expression of certain miRNAs is closely correlated with tumour phenotype, suggesting that miRNAs might function as oncogenes or tumour suppressors [20]. The pleiotropic nature of miRNAs suggests that multiple signalling pathways can be affected by aberrant miRNA expression, thereby significantly directing cancer cell biological behaviour.

Although the role of *SYP* in adrenocortical tumours has been previously reported, the mechanisms involved in abnormal expression of *SYP* in adrenocortical tumours had not yet clarified. In the present study, we aimed to investigate the relationship between aberrant *SYP* methylation status and *SYP* expression in CPA and the mechanisms involved. Our results showed increased expression of *SYP* in CPAs, which is a result of hypomethylation of the *SYP* promoter. Using a miRNA microarray, we found decreased expression of miR-27a-5p in CPAs. By applying gain-of-function and loss-of-function methods and a luciferase reporter assay, we verified that *TET3*, the demethylation enzyme, was the target of miR-27a-5p. Moreover, we determined that the upregulation of *SYP* is associated with DNA demethylation induced by *TET3* in CPA patients. In addition, we found that a miR-27a-5p-*TET3*-*SYP* signal pathway may play a role in the proliferation and cortisol secretion of H295R cells.

## RESULTS

### Expression of *SYP* in human adrenocortical adenoma specimens

To identify the expression of *SYP* in human adrenocortical adenoma, we performed the real time quantitative PCR, western blotting and immunohistochemical staining on 12 adrenocortical adenoma samples from CS and their adjacent normal adrenal cortex tissues.

**Figure 1:**
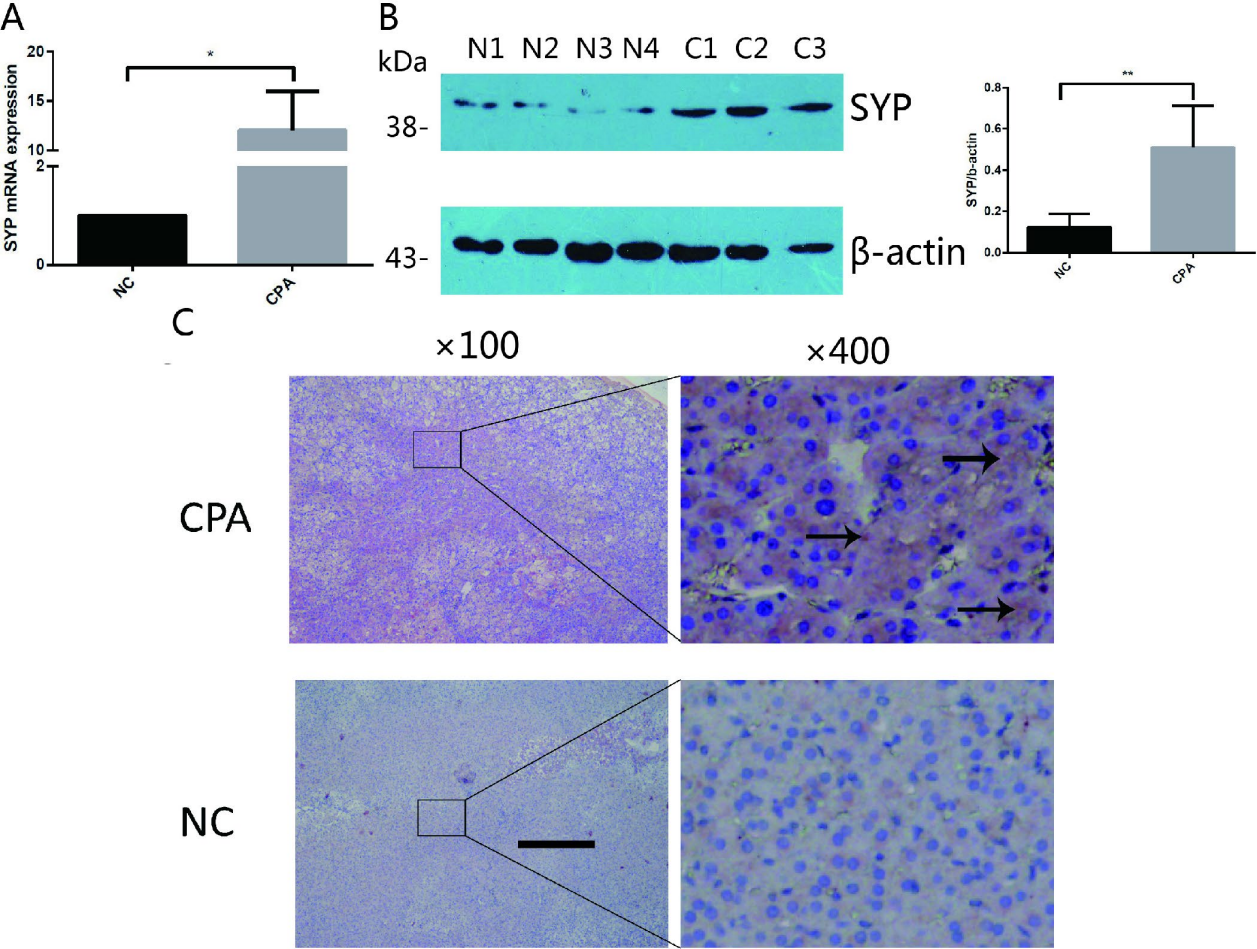
**The expression of *SYP* in CPA.** (**A** and **B**) The relative mRNA levels of *SYP* determined by real-time PCR (**A**) and protein levels of *SYP* determined by Western blotting (**B**) in CPAs and normal adrenal tissues (n=12). (**C**) Immunohistochemisty staining determines the expression of *SYP* in CPAs and normal adrenal tissues. The brown area indicated by arrow is the positive staining of *SYP* in CPAs. Three independent experiments were performed, and representative data are shown. The data represent the mean ± SD. NC, normal control. **p<001.

The qRT-PCR results showed that the expression levels of *SYP* are significantly higher in the adrenocortical adenoma group than in the control group. Compared to the normal adrenal cortex tissues, the expression level of *SYP* mRNA was about 12-fold higher in CPAs (Figure 1A). Likewise, the protein expression levels of *SYP* were also significantly higher in the CPA group compared with the control tissues, as determined by Western blotting (Figure 1B, p<0.05). For the immunostaining, immunoreactivity for *SYP* was present in both groups. However, the intensity of staining for *SYP* was significantly stronger in CPAs than in their adjacent normal adrenal cortex tissues (Figure 1C).

### Epigenetic mechanism of *SYP* up-regulation both *in vitro* and *in vivo*

**Figure 2:**
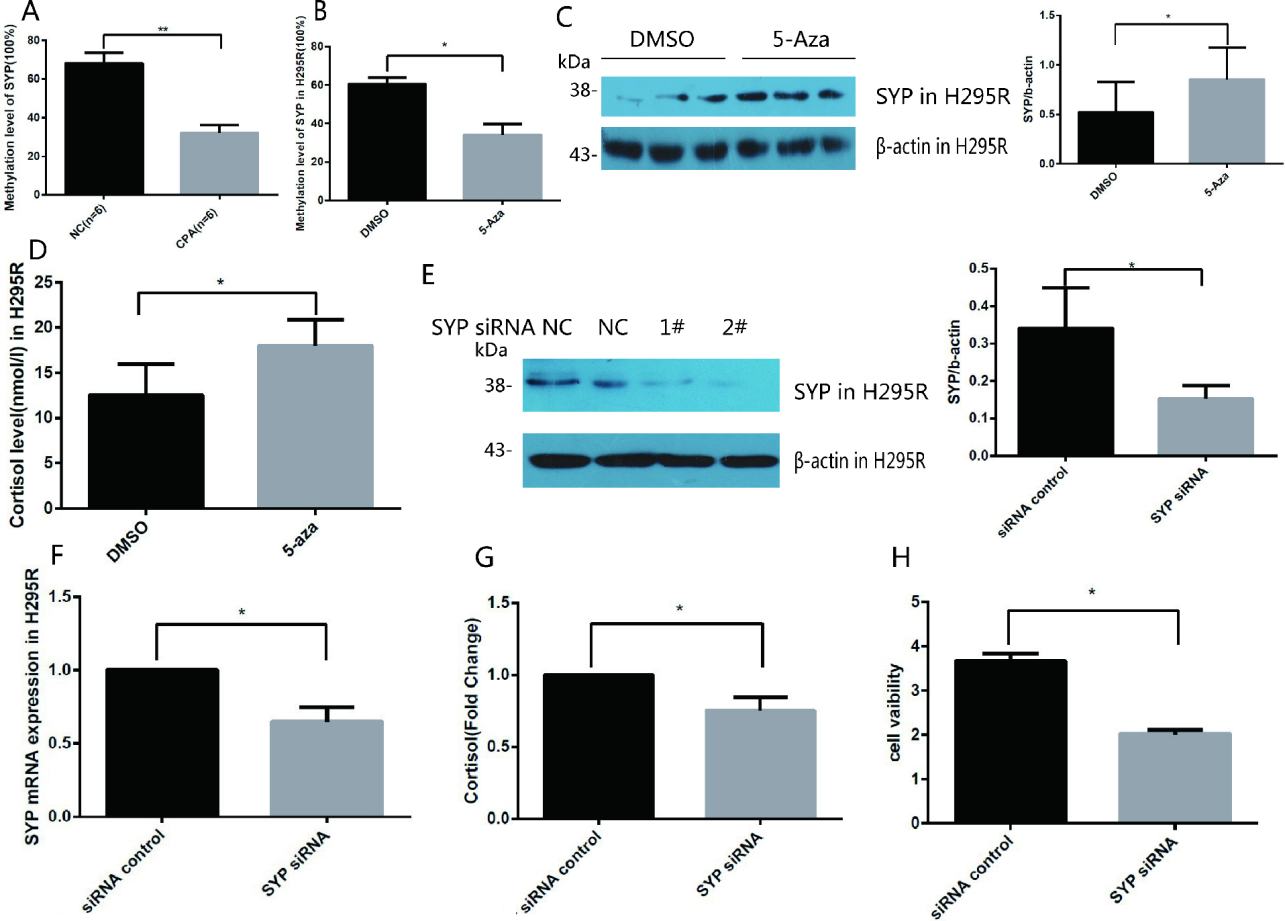
**Methylation of the *SYP* promoter regulates the expression of *SYP* and cortisol secretion and H295R cells proliferation.** (**A**) *SYP* methylation levels in CPA and normal adrenal tissues was determined using EpiTect Methyl II PCR Assay (n=12). (**B**) H295R cells were treated with 5-azaC, *SYP* methylation levels was determined by EpiTect Methyl II PCR Assay. (**C**) H295R cells were treated with 5-aza (5 μm) for 48 h the *SYP* protein level was determined by western blotting. (**D**) H295R cells were treated with 5-aza (5 μm) for 48 h, then cortisol secretion in H295R cell supernatants were measured in cell supernatants by ELISA. (**E** and **F**) Knock-down of *SYP* by siRNA was confirmed by qRT-PCR (**F**) and Western blotting (**E**). (**G**) The H295R cells was treated with *SYP* siRNA or control, then cortisol secretion in H295R cell supernatants were measured by ELISA. (**H**) Knock-down of *SYP* inhibited H295R cell proliferation measured by CCK assay. Three independent experiments were performed, and representative data are shown. Data are shown as mean ± SD. **p<0.01, compared with normal control. *p<0.05, compared with DMSO control.

Next, we explored the molecular mechanism that mediates the upregulation of *SYP* in CPA specimens. Previous studies have demonstrated that there is a CpG island located in the promoter of *SYP*, and the methylation status of the CpG island is related to *SYP* expression. Recently study reported that *SYP* is hypermethylated and decreased in human Alzheimer’s Disease brain tissue [21–23]; therefore, we hypothesised that the upregulation of *SYP* in CPAs is correlated with the promoter of methylation of *SYP*. We applied methylation PCR assay to detect the methylation status of *SYP* in CPA samples and the control samples. The results revealed that in the normal adrenal cortex the promoter of the CpG island was hypermethylated and the methylation rate of CpG sites of *SYP* reached as high as 60.5% whereas the methylation rate of CpG sites in the CPA group was about 34.0% (Figure 2A). To further confirm the promoter hypermethylation in the regulation of *SYP* expression, we treated cultured H295R cells (the adrenocortical carcinoma cell line, the most commonly cell line used to study adrenal tumours, which possesses a steroid secretion and regulation pattern similar to that of primary adrenal cell cultures.) with the DNA methyltransferase inhibitor 5-aza after identifying that the H295R cells had a hypermethylation status in the *SYP* promoter. Our results showed that significantly decreased methylation of the *SYP* promoter (Figure 2B) is associated with increased expression of *SYP* upon 5-aza treatment as shown by western blotting (Figure 2C, *P*<0.05). Thus, we reasoned that DNA methylation of the *SYP* promoter results in different expression of *SYP* both *in vivo* and *in vitro*.

### DNA methylation of *SYP* is involved in cortisol secretion and H295R cell proliferation

To determine whether the overexpression of *SYP* caused by DNA methylation is related to the secretion of cortisol, we measured the secretion of cortisol in cell culture medium after 5-aza treatment in H295R cells. Compared to the control group, the cortisol secretion of the 5-aza treatment group was significantly higher (Figure 2D). To further confirm our speculation that *SYP* plays a crucial role in regulating the expression of cortisol. We successfully transfected *SYP* siRNA 2# into H295R cells to knock down the expression of *SYP*. As shown in Figure 2E, 2F *SYP* expression was significantly downregulated after *SYP* siRNA treatment. Not surprisingly, we found that knock-down of *SYP* results in decreased secretion of cortisol (Figure 2G). Furthermore, the CCK-8 assay showed significant decreases in cell viability with the effective repression of *SYP* in H295R cells (Figure 2H).

### Identification of miRNAs differentially expressed in cortisol-producing adenomas

**Figure 3:**
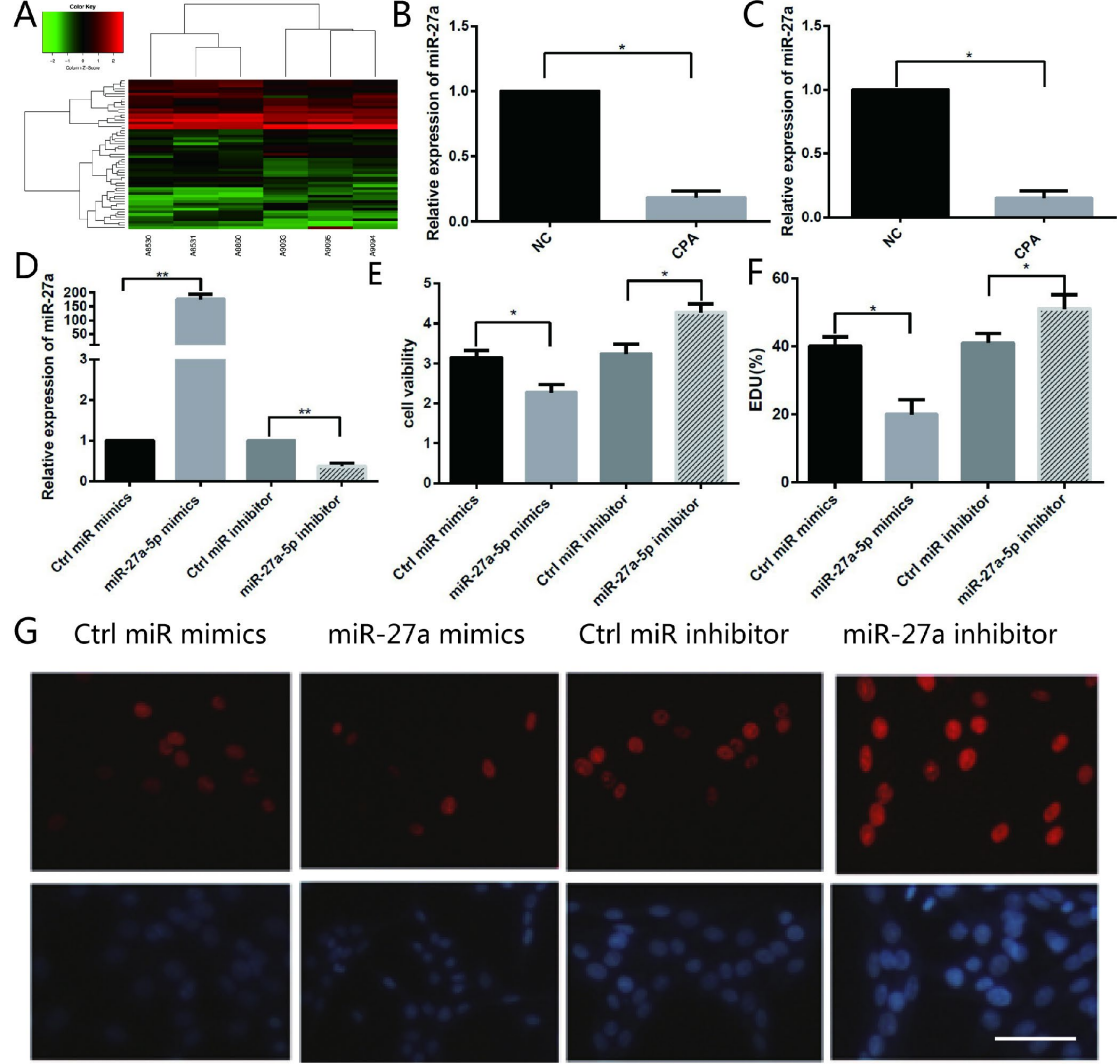
**Different miRNA expression in CPA and normal adrenal tissues and the function of miR-27a-5p on the proliferation of H295R cells.** (**A**) Hierarchical clustering of differentially expressed miRNAs in CPA samples and normal adrenal tissues. (**B**) miR-27a-5p is downregulated about 5.4-fold in CPA in comparison with normal adrenal cortex tissues as indicated by microarray analysis. (**C**) qRT-PCR analysis of miR-27a-5p in CPA samples and their adjacent normal adrenal cortex tissues. Three independent experiments were performed, and representative data are shown. (**D**) Upregulation of miR-27a-5p using miR-27a-5p mimics, or downregulation of miR-27a-5p using miR-27a-5p inhibitor in H295R cells were confirmed by qRT-PCR. (**E**) Cell viability in H295R cells transfected with miR-27a-5p mimics and inhibitor were determined by the CCK-8 assay. (**F**–**G**) Analysis of EdU staining on miR-27a-5p mimic-treated H295R cells. The EdU incorporation rate was expressed as the ratio of EdU-positive cells to total DAPI positive cells. Red, EDU; Blue, DAPI. Magnification, 400×. Three independent experiments were performed, and representative data are shown. NC, normal control. **p<0.01. *p<0.05. NC, normal control.

With the goal of identifying the mechanism involved in dysregulation of *SYP* through methylation, three adrenocortical adenoma samples associated with CPA and their adjacent normal adrenal cortex tissues were chosen to identify the expression of miRNAs. These tissues were then subjected to miRNA microarray analysis, which identified a large number of miRNAs whose expression changed significantly between cortisol-producing adenomas and normal tissues (Figure 3A). According to the results of the microarray analysis, we chose to further investigate miR-27a-5p. The microarray analysis identified that miR-27a-5p is the most downregulated

miRNA in CPAs; its expression decreased 5.4-fold in CPAs (Figure 3B). As expected, the qRT-PCR analysis confirmed that the expression of miR-27a-5p decreased significantly by 9.7-fold (Figure 3C). According to the results of the microarray analysis and qRT-PCR result, we chose to further investigate miR-27a-5p for further investigation and hypothesised that miR-27a-5p might be involved in the development of CPA.

### miR-27a-5p involved in regulating the proliferation of H295R cells

Sharply decreased expression of miR-27a-5p forced us to ask whether miR-27a-5p has a regulatory effect on the proliferation of H295R cells. To identify the role of miR-27a-5p during the proliferation of H295R cells, we used a gain- and loss-of-function approach to overexpress or inhibit expression of miR-27a-5p. Two days after transfection with miR-27a-5p mimics, miR-27a-5p inhibitors, or control oligos, the number of H295R cells were measured by the CCK-8 assay. qRT-PCR results showed that inhibition of miR-27a-5p inhibited miR-27a-5p expression, whereas over-expression induced endogenous miR-27a-5p levels over 184-fold (Figure 3D). The cell viability was significantly reduced by 28% in miR-27a-5p mimic transfected cells compared with NC in the CCK-8 assay, whereas it increased by 31% in miR-27a-5p inhibitor transfected cells compared with NC (Figure 3E). Moreover, we found that the percentage of EDU-positive cells in miR-27a-5p mimic transfected cells decreased significantly and the percentage of EDU-positive cells in miR-27a-5p inhibitor transfected cells showed the opposite results (Figure 3F, 3G). These results indicate that miR-27a-5p plays a negative role in H295R cell proliferation.

### *TET3* is the direct target gene of miR-27a-5p

**Figure 4:**
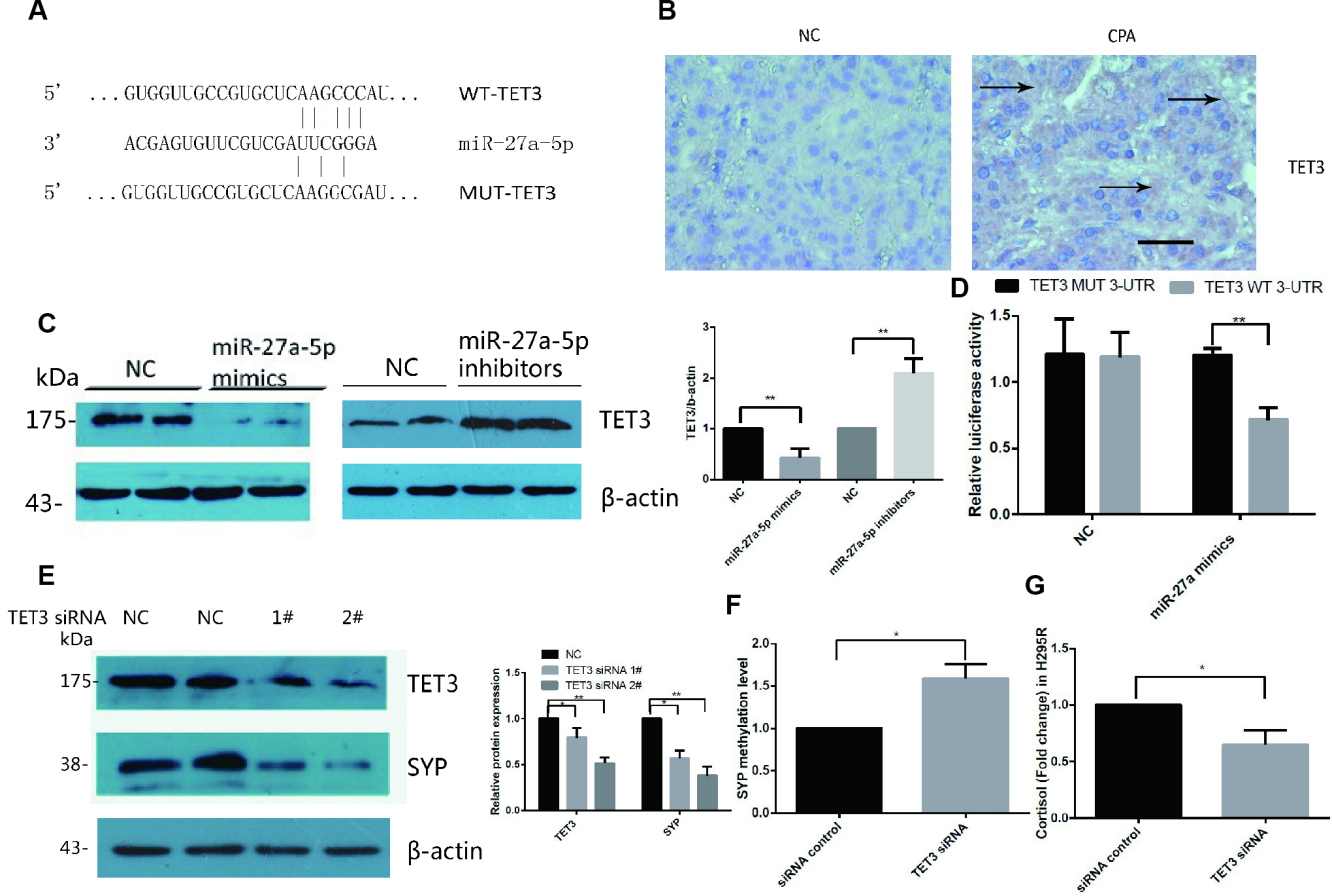
***TET3* is a direct target of miR-27a-5p and *TET3* regulated *SYP* expression and cortisol secretion in H295R cells.** (**A**) RNA22 predicts that *TET3* is a potential target of miR-27a-5p. (**B**) Immunohistochemical staining for *TET3* in CPA and normal adrenal tissue. DAB staining showed that the intensity of staining for *TET3* was significantly stronger in CPAs than in normal adrenal tissues. The brown area indicated by arrow is the positive staining of *TET3* in CPAs. Representative data are shown. (**C**) *TET3* protein levels in H295R cells were assessed by Western blotsin control in miR-27a-5p mimics transfected cells and miR-27a-5p inhibitor transfected cells. (**D**) Luciferase reporter assays were performed using luciferase constructs carrying a WT or mutant *TET3* 3_-UTR cotransfected into H295R cells with miR-27a-5p mimics compared with an empty vector control. Firefly luciferase activity was normalized to renilla luciferase activity. (**E**) Western blot analysis of protein levels in H295R cells transfected with *TET3* siRNA (siRNA 1#,2#) or scrambled control for 48h. (**F**) Methylation level of *SYP* promoter was analysis by EpiTect Methyl II PCR Assay in H295R cell transfected with *TET3* siRNA or control. (**G**) Cortisol secretion were measured in cell supernatants by ELISA from H295R cell transfected with *TET3* siRNA or control. Error bars represent SD. Three independent experiments were performed, and representative data are shown. The data represent the mean SD. NC, normal control. **p<0.01. *p<0.05.

miRNAs exert their function by regulating the expression of their downstream target genes [24]. We utilized several bioinformatic target prediction algorithms to search for targets related to miR-27a-5p. *TET3* was revealed to be a potential target of miR-27a-5p (Figure 4A). Accordingly, we evaluated the expression levels of *TET3* in the tissue samples used for immunohistochemistry. The intensity of staining for *TET3* was significantly stronger in the CPAs than in their adjacent normal adrenal cortex tissues (Figure 4B). Furthermore, we detected downregulation of the *TET3* protein in miR-27a-5p mimic transfected cells and upregulation of the *TET3* protein in miR-27a-5p inhibitor transfected cells by Western blot analysis (Figure 4C).

To further determine whether miR-27a-5p directly binds *TET3* mRNA and regulates its expression, a luciferase reporter construct containing the wild-type or mutant 3′-UTR coding sequences for *TET3* was generated and introduced into miR-27a-5p mimics and H295R cells. Overexpression of miR-27a-5p significantly decreased the relative luciferase activity of the WT-3′-UTR of *TET3* reporter plasmids, but when the miR-27a-5p seed sequence in *TET3* mRNA 3′-UTR was mutated, the inhibitory effect of miR-27a-5p on the relative luciferase activity was abrogated (Figure 4D). Negative control miR-27a-5p mimics did not affect the wild-type or mutant constructs, confirming the specificity of the action. The results confirmed that *TET3* is the direct target of miR-27a-5p.

### *SYP* up-regulation is associated with *TET3*

The DNA methylation/demethylation status was controlled by the *TET* family of methylcytosine dioxygenases. We hypothesised that *TET3* is associated with *SYP* hypomethylation in CPAs. Thus, we investigated the effect of *TET3* knock-down by siRNA in H295R cells. The western blot results confirmed that *TET3* was significantly downregulated when transfected with siRNA 2# (Figure 4E). As expected, *SYP* was significantly downregulated after being transfected with *TET3* siRNA in H295R cells for two days (Figure 4E) and the methylation level of it significantly increased (Figure 4F). Secretion of cortisol was decreased at the same time (Figure 4G).

## Discussion

**Figure 5:**
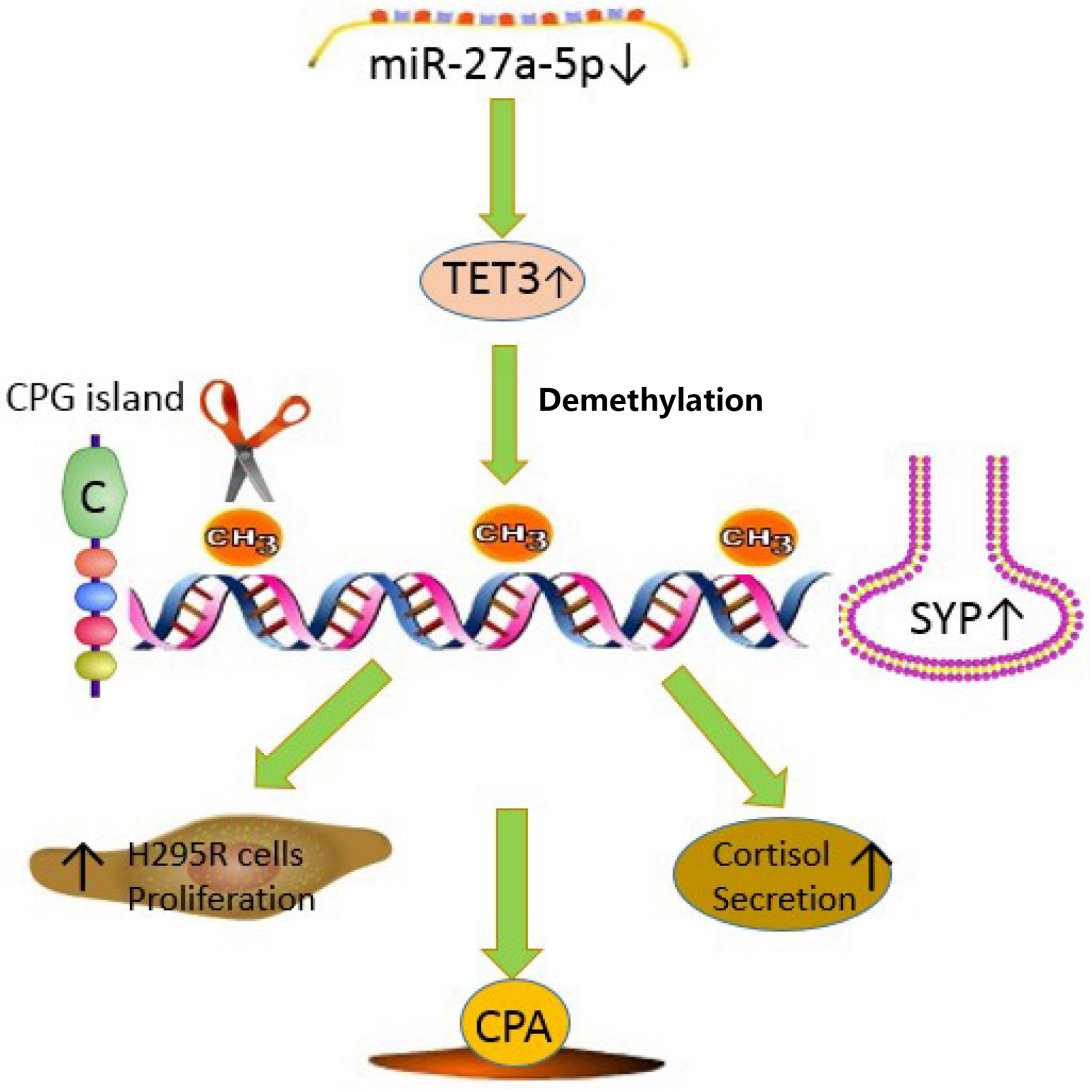
**The mechanism diagram about miR-27a-5p-*TET3*-*SYP* signal pathway in adrenocortical adenomas causing CPA.** miR-27a-5p suppresses *SYP* through epigenetic repression by targeting *TET3* in adrenocortical adenomas causing CPA. miR-27a-5p-*TET3*-*SYP* signalling pathway may play a key role in CPA progression, H295R cell proliferation and cortisol secretion.

In the present study, we analysed the mechanism of the miR-27a-5p-*TET3*-*SYP* signal pathway in adrenocortical adenomas causing CPA dysregulation (Figure 5). This analysis resulted in several novel findings that shed light on the molecular mechanism of CPA. Firstly, we used immunohistochemistry, western blotting and qRT-PCR to identify *SYP* expression signatures in CPA tissues and normal adrenal cortex tissues and found that *SYP* is significantly upregulated in the CPA tissues compared to the control group. Next, we showed that *SYP* is regulated by DNA methylation both *in vitro* and *in vivo* and the overexpression of *SYP* is related to the secretion of cortisol and cell proliferation. Then, we used a microRNA array to profile the microRNA expression signatures in CPA and normal cortex tissues and found that miR-27a-5p is one of the most significantly down-regulated microRNAs in the CPA group. We showed that miR-27a-5p plays a pivotal role in the regulation of function of H295R cells. Finally, we found that *TET3* is the direct target of miR-27a-5p and confirmed that *SYP* up-regulation is associated with hypomethylation induced by *TET3* in CPA.

Recently, increasing evidence has clarified that *SYP* is ubiquitously expressed in adrenocortical tumours [12]. Nevertheless, there was no study about the the mechanisms involved in *SYP* aberrantly expressed in adrenocortical tumours. Moreover, further examination of *SYP* expression among adrenal disorders has not been extensively performed. Hence, we demonstrated that *SYP* expression was strikingly higher in the CPA than their adjacent normal adrenal cortex tissues by using qRT-PCR and immunohistochemistry. Western blotting, using the same antibodies as in the immunohistochemistry, also demonstrated a significantly increased expression of *SYP* in CPAs.

Recent studies have shown that a common epigenetic aberration in cancer involves deregulated DNA methylation such as CpG island hypermethylation that leads to gene silencing of specific tumour-suppressor genes [25–27]. Interestingly, a global hypomethylation has recently also been postulated to be an important contributor to tumourigenesis such as hepatocellular carcinoma [28]. Moreover, there are studies that demonstrated that primary and metastatic adrenocortical carcinoma samples have a global hypomethylation pattern compared with normal and benign adrenocortical tissue samples, whereas other studies clarified that some genes involved in cell cycle regulation, and apoptosis in the development of adrenal showed significant and frequent hypermethylation [29–31]. In addition, Brandi et al. found that aldosteronomas are globally hypomethylated and *CYP11B2* is overexpressed, which is associated with hypomethylation in these tumours [15,32]. However, only a few reports have addressed DNA methylation in CPA and whether DNA methylation affects *SYP* expression in CPA has remained unknown. Therefore, we used methylation PCR assay to detect the aberrant methylation of *SYP* in CPA and the control group. We confirmed the presence of hypomethylation of the *SYP* promoter in CPA compared with normal adrenal cortex. Moreover, demethylation treatment of the adrenocortical carcinoma cell line H295R using 5-aza resulted in increased expression of *SYP*, which also supports the finding that *SYP* expression is regulated by an epigenetic mechanism. These findings provide the first evidence of a mechanism by which *SYP* is upregulated in CPA through epigenetic regulation of this gene.

A previous study reported that *SYP* mRNA levels showed a positive correlation with mRNA levels of *CYP17A1* encoding 17a-OH [12]. They suggested that *SYP* expression in adrenocortical cells may be involved in some aspects of adrenal function such as transport or secretion of glucocorticoids. On the other hand, the corticosteroid hormone cortisol is an important determinant of blood pressure and cardiovascular risk [33]. For this reason, we detected whether the over-expression of *SYP* caused by DNA hypomethylation is related to the secretion of cortisol. After demethylation treatment of the adrenocortical carcinoma cell line H295R with 5-aza, the secretion of cortisol increased compared with the control group. Then, we knocked-down the expression of *SYP* in H295R cells and demonstrated that the effect of *SYP* knock-down decreased the secretion of cortisol. Moreover, the finding that *SYP* inhibits H295R cell proliferation was also confirmed.

Numerous evidences have shown the association of aberrantly expressed miRNAs with tumour development and progression [34–36]. Several studies have reported the different miRNA expression profiles in various types of adrenocortical adenomas compared with normal adrenal gland tissues [37–39]. Nevertheless, there have not been many studies directly comparing CPA and normal tissues using a miRNA microarray. In our present study, we established the specific miRNA expression profile in CPA compared with normal adrenal cortex tissues, and confirmed that the expression levels of miR-27a-5p were dramatically downregulated in CPA samples and involved in regulating the proliferation of H295R cells.

*TET1*, *TET2*, and *TET3*, the ten eleven translocation (*TET*) family of methylcytosine dioxygenases, can transform 5-methylcytosine (5mC) into 5-hydroxymethylcytosine (5hmC), and eliminate extant methylation labels in cells [40–42]. Specifically, the 5hmC level, which has a close connection with the gene expression of the *TET* family of methylcytosine dioxygenases, is raised in differentiated cells and sharply decreased in numerous cancer types, suggesting that the 5hmC level is negatively correlated with tumour progression [43,44]. The DNA methylation/demethylation status controlled by the *TET* family of methylcytosine dioxygenases, may impact the development of tumours. *TET3* in mouse is reported to contribute to zygotic epigenetic reprogramming and global demethylation of the male pronucleus. During vertebrate neurogenesis, *TET3* also plays an important role in enrichment of 5hmC at neurodevelopmental genes. Recently, a report showed that mutations of the isocitrate dehydrogenase (*IDH*) genes *IDH1* and *IDH2* can inactivate methylcytosine dioxygenase activity, resulting in DNA hypermethylation in acute myeloid leukemia cells [45]. This conclusion further enhances the outlook that TET methylcytosine dioxygenase plays a critical role in aberrant epigenetic regulation in cancer progression. Moreover, a study recently found that upregulation of *ARL4C*, due to DNA hypomethylation induced by TET upregulation, promotes tumourigenesis of lung squamous cell carcinoma [46]. In this study, we demonstrated that miR-27a-5p can directly regulate expression levels of *TET3*, suggesting that *TET3* may serve as a major gene target in mediating the effect of miR-27a-5p in H295R cells proliferation and cortisol secretion. Our results further demonstrated that knock-down of *TET3* can reduce the expression level of the *SYP* gene in H295R cell. Moreover, knock-down of *TET3* results in decreased secretion of cortisol.

In this study, we just had twelve CPAs. The number of clinical samples is far from enough to demonstrate the conclusion. Obviously, more clinical samples will be needed to clarify the underlying mechanism.

In summary, our present study revealed that the miR-27a-5p-*TET3*-*SYP* signalling pathway may play a key role in CPA progression, H295R cell proliferation and cortisol secretion. *SYP* acts as a crucial regulator of adrenal hyperfunctions and the mechanism involved was also revealed. In addition, we clarified that *TET3* is the direct target gene of miR-27a-5p. The overexpression of *SYP* is related to its hypomethylation of the CpG sites induced by *TET3* in CPA. To the best of our knowledge, our results show for the first time the role of miR-27a-5p and *SYP* in the expression of cortisol secretion and proliferation of H295R cells. This is also the first time to reveal the mechanism of up-regulation of *SYP* expression in CPA. Therefore, the study suggested that miR-27a-5p, *TET3* and *SYP* could be potent therapeutic agents. Investigating the role of the miR-27a-5p-*TET3*-*SYP* signalling pathway on adrenocortical adenomas will provide new insight into adrenocortical adenomas, and based on this understanding, future drugs targeting specific genes may be developed to treat CPA.

## Materials and Methods

### Tissue samples

Adrenocortical tissue sample collections were approved by the Ethics Committee of the Second Xiangya Hospital, Central South University, complied with the Declaration of Helsinki and written informed consent was obtained from all participants in our experiments.

Twenty-four adrenal tissue samples (12 adrenocortical adenomas from patients with adrenal Cushing’s syndrome, 12 from paired adjacent normal adrenal cortex) were obtained at surgical resection and immediately snap-frozen and stored at −80°C. The diagnosis of CPA was based on a typical clinical manifestation, increased serum cortisol levels with abnormal diurnal rhythm accompanied by decreased serum ACTH levels, increasing 24 h urinary-free cortisol, an un-suppressible result on the dexamethasone challenge test, and computed tomography results revealing unilateral adrenal tumours in all the patients. Moreover, the pathologic study confirmed the diagnosis. The clinical characteristics are summarized in Table 1.

**Table 1:** The clinical characteristics of CPA patients.

		**Patients**
N		12
Sex		
Male		5(41.7%)
Female		7(58.3%)
Age		44.92±9.38
Body mass index(kg/m^2^)		26.71±3.38
8:00 serum cortisol(nmol/l)		628.98±198.56
16:00 serum cortisol(nmol/l)		571.47±275.78
24:00 serum cortisol(nmol/l) 24 h urinary free cortisol(nmol/24h) 8:00 plasma ACTH(ng/l)		531.24±234.29 1909.39±1059.34 9.04±9.17

ACTH, Adrenocorticotropic hormone

### Reagents

Dimethyl sulfoxide (DMSO) and 5-Aza-2′-deoxycytidine (5-Aza) were purchased from Sigma-Aldrich (St Louis, Missouri, USA). DMEM and fetal bovine serum were purchased from Gibco BRL Co. (Grand Island, New York). Lipofectamine 2000 was purchased from Invitrogen Co. (Carlsbad, California, USA). The antibody for β-actin was purchased from Abgent Inc. (San Diego, California, USA). Antibodies for *SYP* and *TET3* were purchased from Abcam (Cambridge, England). Maxima SYBR Green/ROX qPCR Master Mix was purchased from Genecopoeia and all of the primers used in this research were purchased from Genecopoeia. The related secondary antibodies and the ECL detection kit were purchased from Santa Cruz Biotechnology, Inc. (Santa Cruz, California, USA). *SYP* and *TET3* siRNA oligos and control siRNA oligos were purchased from Ribobio (Guangzhou, China). miR-27a-5p mimics, inhibitors and their control oligos were purchased from Ribobio. DAPI was purchased from Solarbia (Beijing, China). The EdU kit was purchased from Ribobio. The CCK8 kit was purchased from 7 sea biotech (Shanghai, China).

### Cell culture treatment and transfection

The adrenocortical carcinoma cell line H295R was obtained from the National Platform of Experimental Cell Resources for SciTech (Beijing, China). Cells were maintained in DMEM:F12 medium (Gibco, Life Technologies, Grand Island, New York, USA) supplemented with 2.5% of NuSerum (BD Biosciences, Bedford, Massachusetts, USA), 1% penicillin/ streptomycin (Gibco) and 1% insulin–transferin–selenium culture supplement (BD Biosciences) at 37°C in 5% CO_2_ infusion and humidified air and the medium was refreshed every 2 days.

After passaging for 12 hours, cells were treated with 5-aza at 5 uM (Sigma-Aldrich) and vehicle for 24 and 48 hours. The medium was replaced every 24 hours. Total protein and DNA was extracted after 48 hours of treatment and analysed for *SYP* protein and methylation expression level. The experiments were repeated three times. For transient transfection of *SYP* siRNA oligos, a combination of oligos (50 nM) and Lipofectamine 2000 was mixed following the manufacturer’s instructions and added to cells in 6-well plates at a density of 2 × 10^5^ cells per well.

### Measurement of cortisol

Cortisol secreted into the culture media was measured by competitive ELISA using commercial EIA kits (Cayman Chemical) according to the manufacturer’s instructions.

### Western blot analysis

Western blot analysis was carried out for detection of *SYP*, *TET3* or β-actin protein levels as previously described. Briefly, 30 μg of protein from each cell layer extract was loaded onto the same SDS PAGE and then transferred to a PVDF membrane. After blocking with 5% non-fat milk, the membrane was incubated with *SYP*, *TET3* and β-actin overnight at 4°C. The next morning, the membrane was washed with PBS three times every ten minutes. The membrane was then incubated with appropriate secondary antibody at 1:2000 dilution in 2% non-fat milk for 1 h. Blots were processed using an ECL kit, exposed to film and then analysed by densitometry.

### miRNA microarray analysis

Total RNA was extracted from cortisol-producing adenoma tissue samples and normal adrenal cortex tissue samples. All samples were assessed for enriched miRNA using an agilent 2100 Bioanalyzer. miRNAs were then labelled using the FlashTag Biotin RNA labelling kit (Affymetrix, Santa Clara, CA, USA) following the manufacturer’s protocol and then hybridized to Affymetrix GeneChip miRNA 1.0 microarrays (Affymetrix). Each miRNA was measured in duplicate.

### Gene expression estimated using qRT-PCR

Total RNA was extracted from cortisol-producing adenoma tissue samples and normal adrenal cortex tissue samples, and cDNA was prepared. For analysis of miR-27a-5p expression, reverse transcription and quantitative reverse transcription-polymerase chain reaction (qRT–PCR) were carried out using the primer for human miR-27a-5p (Genecopoiea, MmiRQP0538) and U6 snRNA (Genecopoiea, China) according to the manufacturer’s instructions. *SYP* (HQP017850) and GAPDH (MQP027158) gene primers were purchased from Genecopoiea, and their mRNA expression was also measured by qRT–PCR amplification. Relative quantification was calculated through the 2^-△△CT^ method.

### Gene specific DNA methylation determination

Total genomic DNA was isolated from cortisol-producing adenoma tissue samples, normal adrenal cortex tissue samples and H295R cells using QIAamp DNA mini kit (Qiagen, Germany). Genomic DNA was isolated using the binding column according to the manufacturer’s instructions. Gene specific DNA methylation was determined by using an EpiTect Methyl II PCR Assay (Qiagen, Germany) and methyl primer synaptophysin (catalog# EPHS115006-1A). We evaluated the methylation level of *SYP* according to the manufacturer’s instructions.

### Immunohistochemistry

Paraffin-embedded 4-mm-thick specimens were dewaxed in turpentine and rehydrate through decreased concentrations of ethanol. Antigen retrieval was not performed. Endogenous peroxidase activity was blocked by using 3% H_2_O_2_ in methnal for 10min and then soaked with phosphate buffered saline (PBS) (pH 7.2–7.4) three times for 5 min. The sections were then pre-incubated with 0.25% trypsin-EDTA for 10 min to block non-specific antigens. The tissue sections were allowed to react overnight at 4°C with anti-*SYP* (dilution 1/100; Abcam) *TET3* (dilution 1/200, Abcam) antibodies. The slides were then incubated at room temperature for 1h, rinsed with PBS three times and incubated with appropriate biotinylated secondary antibodies for 30 min. For the quantification of immunohistochemistry, 10 fields of each section were viewed and analysed with an image analysis program (Bioquant, Nashville, TN).

### Plasmid constructs and luciferase reporter assay

For functional analysis of miR-27a-5p, segments of the *TET3* 3’-UTR, including the predicted miR-27a-5p binding sites, were amplified using PCR and cloned into the PmeI and XbaI restriction sites of the luciferase reporter vector pmirGLO (Promega, Madison, WI, USA), resulting wild-type *TET3* 3’UTR (WT-*TET3*-3’UTR). The *TET3* mutants for the miR-27a-5p seed regions were prepared using the QuikChange Site-Directed Mutagenesis Kit (Stratagene, San Diego, CA, USA) to get mutant *TET3* 3’UTR (MUT-TET3-3’UTR). Sequences of the PCR and mutagenic primers are shown in (Table 2). H295R cells were transfected with either WT-*TET3*-3’UTR or MUT-*TET3*-3’UTR and miR-27a-5p mimics or control for 48 h. Luciferase activities were detected with the luciferase assay system (Promega). 

**Table 2:** Nucleotide sequences of primers for WT and mutant reporter plasmids.

**Gene**	**Primer sequence (5′ to 3′)**
WT *TET3*	Forward: 5′ TAGTCTAGAAGAGGTGAGTCAAGAGGCAGTC 3′
	Reverse: 5′ GGCCGGCCACGCAACAGGCAGGGAAA 3′
Mutant *TET3*	Forward: GCGGTGTGGTTGCCGTGCTCAAGGCGATGCTGATTTGTAC
	Reverse: CGCCACACCAACGGCACGAGTTCCGCTACGACTAAACATG

### Statistical analysis

The results of the experiments are presented as means ± SD, and analysis was performed with Statistical Product and Service Solutions (SPSS) software (version 19.0). Comparisons between values of more than two groups were evaluated by an analysis of variance (one-way ANOVA). A level of *p*<0.05 was considered statistically significant. All experiments were repeated at least three times, and representative experiments are shown in the Figures.
